# A Treatise the Diseases of the Liver and on Bilious Complaints

**Published:** 1833-10-01

**Authors:** 


A Treatise on the Diseases of the Liver, and on Bilious Complaints; with Observations on the Management of the Health of those who have returned from Tropical Climates, and on the Diseases of Infancy. By *George Hamilton Bell*, Fellow of the Royal College of Surgeons, Edinburgh; late residency Surgeon, Tanjore. One vol. 8vo. pp. 152. Edin. 1833.

Mr. Bell is already favourably known to his professional brethren, by his work on cholera; and his long residence in India must have furnished him with ample materials for the present publication. It is certainly remarkable, as Mr. Bell observes, that so few of our Indian practitioners have recorded the fruits of their experience for the benefit of their successors. The great work of Annesley is a splendid exception; but its magnitude renders it almost useless to the great mass of medical men in our Asiatic possessions. Concise and succinct treatises on particular diseases are desiderata yet in Oriental medical literature, and Mr. Bell has here attempted to fill up one of the lacunæ from notes taken at the bedside of sickness, and subsequent reflexion on the cases there presented to his view. It is not to be supposed that the subject of this volume is only interesting to those who are destined for inter-tropical practice.

"Judging from what may almost be called the fashionable prevalence of bilious affections, one would be apt to suppose that something like an intertropical tendency to liver complaints had been imported into this country. Such a supposition, indeed, is not without plausibility. The sources of what are usually termed hereditary diseases may often be traced to some misfortune, neglect, or imprudence in a predecessor. Thus the habits of living of a father, a grandfather, or even of some more remote ancestor, or a cold which he has neglected, may have engendered the gout, or the consumption, under which his descendants suffer. So it is well known that every one who has been much exposed to a hot climate acquires a predisposition to hepatic affections; and when we remember the number of our countrymen, or of their descendants, who annually return from the intertropical possessions of Britain, labouring under the diseases of the climate, and become fathers of families; or who themselves suffer during the remainder of their lives under the morbid affections which they have brought with them, we shall have no reason to be surprised at the diffusion and very general prevalence of diseases, which have not hitherto been regarded as indigenous in the temperate zones."—*Pref.* vii.

Although we are fully persuaded that many cases of supposed liver complaints are, in reality, affections of the stomach and intestines, yet there can be little doubt that such an organ as the liver must be very frequently disturbed in function, and even altered in structure, by the multitude of causes, moral and physical, which daily operate among all classes of society in this country.

We shall pass over some theoretical speculations on the nature of inflammation, and come at once to the practical. Mr. B. thinks himself justified in subdividing phlegmonous inflammation of the liver into what he terms sero-phlegmon, puro-phlegmon, and muco-puro-phlegmon. The first makes its attacks on naturally colourless parts—the second, on those vascular parts which, in the healthy state, have red blood circulating, and in which the pain is obtuse, and there is a tendency to the deposition of pus —the third, attacks mucous surfaces, and is accompanied by pain, heat, swelling, &c. terminating in changed secretion, and ulceration. Our readers will readily perceive that the above terms convey what we mean by inflammation of the peritoneal covering, the parenchyma, and the ducts, &c. of the liver. Were it not that every author must do something to correct (sometimes to confuse) the nosological portions of our science, the old mode of arranging hepatitis might have been continued.

I. *Sero-hepatitis.* The following is the concise symptomatology presented by Mr. Bell.

"SYMPTOMS.—Those which are peculiar to this affection, in whatever part of the liver it is seated, are, 1. A sudden attack of excruciating pain in the region of the liver, often so severe that the weight of the clothes is insupportable. 2. High febrile symptoms, compelling the patient immediately to confine himself to bed. 3. The stomach is irritable, and the biliary secretion is generally increased. 4. The patient cannot lie on the left side." 12.

Mr. Bell makes some remarks on the means of distinguishing membranous inflammation of the liver from pleural inflammation in the contiguous portions of the chest; but the distinction is of little importance, since the treatment will be nearly the same. The following passage, however, is worth quoting.

"But it is not only *inflammatory* affections of the colon which may mislead as to existence of hepatitis. Many who have been exposed to tropical climates, or other causes of hepatic affections, acquire a morbid sensibility of the liver, or of the great gut, which sometimes becomes so intense as to give rise to nearly all the symptoms of acute sero-hepatitis. I have seen cases in which this morbid sensibility has been treated as inflammation, the symptoms being of course aggravated by antiphlogistic remedies. We must in such circumstances be guided by the state of the pulse and the skin. The febrile symptoms seldom run high when the pain referable to the liver arises merely from morbid irritability; and it is valuable as a discriminating symptom, that, though the pain of irritability is much aggravated by slight pressure, it is relieved when the pressure is increased. As the treatment of inflammation and morbid irritability is very different, it is of the greatest consequence not to mistake the one for the other." 15.

The etiology of this form of hepatitis, especially in India, is chiefly connected with severe exercise in the heat of the day, together with exposure to wet, as in shooting, &c. Exposure to cold or damp, in all climates, indeed, will sometimes produce sero-hepatitis. In this species it is seldom necessary to put the system under the influence of mercury. The common depletory measures used in pleurisy are proper here. There is, however, an irritability of the stomach attending sero-hepatitis, which requires a peculiar modification of treatment.

"Severe retching is not only a source of injury to the inflamed membrane, but a serious interference with the exhibition of remedies. The above, therefore, is an object of primary importance. It will sometimes happen that a full bleeding quiets the stomach, and enables us at once to exhibit purgatives. Should this not be the case, a scruple of calomel, while it produces the most beneficial effects throughout the whole gastro-enteritic mucous membrane, acts like a charm in allaying irritability of the stomach. A large dose of calomel, therefore, should be prescribed; and its effects may be forwarded by the application of a large sinapism over the stomach. And effervescing saline draughts, while they are agreeable to the patient, are soothing to the stomach. Should these means be ineffectual in putting an end to the retching, leeches, and a blister to the scrobiculus cordis, must be resorted to." 21.

II. Puro-Hepatitis. Inflammation and suppuration in the substance of the liver produce great havoc among our European settlers in India.

" *Symptoms.* The condition of the hepatic vessels which leads to suppuration in the substance of the liver, seems to be so little different from their usual state (at least so far as is indicated by symptoms), that very frequently the first intimation which a patient has of serious disorder of the system, is what is too often to be reckoned proof of the formation of an abscess. He is attacked with a shivering fit, which is followed by an irregular hot stage, ending in profuse clammy perspiration. Even after this there may be no symptom pointing out the destruction which is going on in his liver. The patient suffers from irregular feverish symptoms, and has the impression that something very wrong is taking place; but neither he, nor probably his medical attendant, is aware that he is stricken with a mortal malady. As the case advances, there are occasional severe shivering fits, and distressing night sweats—the pulse rises—the tongue is furred—and from the appearance of the patient's countenance, it is evident that he is labouring under some great internal disease. Still there may be no symptom referable to the liver; great derangement of the bowels ensues, and there is much suffering from dyspeptic symptoms. In some instances there are severe spasms in the diaphragm, and violent tenesmus. After some days (or it may be even weeks) the patient is attacked with low delirium, and dies as if from effusion in the brain. This is an extreme case.

In the less obscure cases there are fulness, weight, and uneasiness in the right side, increased on pressure. There is pain in the right shoulder, or in the back; there is a dry cough, the stomach is disordered, and the bowels much deranged; and though the bowels may not be materially affected, there are alternate aguish and feverish symptoms. There is urgent thirst, the tongue is furred, and the urine high-coloured, depositing a lateritious or pinky sediment. There is great risk that these symptoms will soon be followed by decided indications of the formation of an hepatic abscess." 31.

These cases rarely require or bear strong depletion. Leeches or cupping are all that are, in general, proper.

" In puro-hepatitis, no time is to be lost in endeavouring to produce salivation; an effect of mercury which is the more satisfactory, as proving that the cure is within our reach. For in cases in which abscess exists, although the mouth sometimes becomes ulcerated, under the use of mercury, true ptyalism does not take place.

Calomel is the usual form in which mercury is prescribed in this disease in India; this preparation in large doses being generally considered the most rapid in producing the constitutional effects of the medicine, and it has the advantage of not acting as a purgative when taken in large quantities. The ordinary method in puro-hepatitis is to administer calomel in scruple doses alone, or in combination with opium or hyosciamus, every six or eight hours; mercurial ointment being, at the same time, rubbed in on the thighs or abdomen." 46.

III. Chronic Hepatitis. This is the disease which comes more frequently under our attention in this climate. The following is the brief symptomatology given by Mr. Bell.

"*Symptoms.* The symptoms proper to this disease, in whatever portion of the coat of the liver it is situated, are weight, and a feeling of fulness in the right hypochondrium ; shiverings, and occasional and irregular feverish attacks, with heat of skin, and thirst; a foul brown or white tongue, with prominent papillæ; the pulse is generally hard, but not much accelerated; an increased and unhealthy condition of the biliary secretion; irritability and derangement of the stomach and bowels, and not unfrequently dysenteric symptoms ; a sallow or jaundiced skin ; urine scanty and high-coloured; pain over the right clavicle, or in the direction of the right scapula, with inability to lie comfortably on the left side ; sleep disturbed." 68.

In respect to treatment, it may be remarked that topical bleeding is generally necessary, and that repeatedly, together with blisters to the side. The bowels must be constantly attended to ; and mercury must be slowly introduced until slight ptyalism is induced. The other means recommended by Mr. Bell are those in general use,

Many judicious observations are made on functional disorders of the liver —on jaundice—disorders of tropical valetudinarians—diseases of children, &c. for which we must refer to the work itself. We cannot say that in this volume, Mr. Bell has taken such a commanding attitude for originality as in his Essay on Cholera : nevertheless the publication will be very useful, especially to those who are about to embark for our tropical possessions in the East.

